# Parabrachial calcitonin gene-related peptide neurons sex-specifically modulate behavior in anxiety assays during alcohol withdrawal

**DOI:** 10.3389/fphar.2025.1750956

**Published:** 2026-02-12

**Authors:** C. E. Van Doorn, J. B. Tyree, A. A. Jaramillo

**Affiliations:** 1 University of Kentucky, College of Pharmacy, Lexington, KY, United States; 2 Department of Pharmaceutical Sciences, Lexington, KY, United States; 3 Vanderbilt University School of Medicine, Nashville, TN, United States; 4 Department of Molecular Physics & Biophysics, Nashville, TN, United States; 5 Vanderbilt Brain Institute, Nashville, TN, United States; 6 Vanderbilt Center for Addiction Research, Nashville, TN, United States

**Keywords:** alcohol, alcohol withdrawal, anxiety, bed nucleus of the stria terminalis (BNST), CGRP- calcitonin gene-related peptide, negative affect, parabrachial nucleus (PBN), stress

## Abstract

Parabrachial nucleus (PBN) neurons expressing Calcitonin Gene-Related Peptide (CGRP) modulate fear- and anxiety-like behavior. It is unknown if PBN(CGRP) neurons play a role in anxiety during withdrawal from alcohol or after repeated stress. First, to investigate the anxiogenic role of activating PBN(CGRP) neurons in naïve conditions, *Calca* ^CRE^ female and male mice expressing CRE-dependent hM3D (Gq) DREADDs in the PBN were tested on the elevated plus maze (EPM). PBN(CGRP) neurons drive phasic activity in the bed nucleus of the stria terminalis (BNST) that synchronizes to anxiety-like behavior. Therefore next, a transsynaptic anterograde AAV-based strategy was used in C57BL/6J female and male mice to activate BNST neurons innervated by the PBN projections (BNST^PBN^) during EPM. Additionally, the quantity of PBN(CGRP) neurons and CGRP-innervated BNST cells was measured in experimentally-naïve CGRP-DTR^GFP^ female and male mice, to investigate baseline sex-differences. Next, to investigate the impact of PBN(CGRP) inhibition on anxiety-like behavior following chronic intermittent ethanol vapor exposure (CIE), *Calca* ^CRE^ female and male mice expressing CRE-dependent hM4D (Gi) DREADDs in the PBN were tested in EPM during acute withdrawal. Additionally, mice were exposed to repeated forced swim stress (FSS) paired with PBN(CGRP) inhibition followed by testing in the novelty suppressed feeding task (NSFT), to investigate the role of PBN(CGRP) on anxiety-like behavior after stress in protracted withdrawal. Activating PBN(CGRP) or BNST^PBN^ neurons did not change behavior in EPM in either sex. Total PBN(CGRP) and CGRP-innervated BNST cells did not differ between females and males. In acute withdrawal, inhibiting PBN(CGRP) neurons sex-specifically decreased total distance travelled and average speed in EPM. In protracted withdrawal, inhibiting PBN(CGRP) neurons during FSS disrupted the increase in immobility across sessions in females with no effect in males. A history of inhibiting PBN(CGRP) neurons during FSS increased anxiety-like behavior during NSFT, with implications for decreased motivation. Altogether, the data report that in alcohol withdrawal females and males differentially respond to PBN(CGRP) manipulations albeit they are both sensitive to the negative affect induced by repeated PBN(CGRP) manipulations during stress.

## 1 Introduction

Despite the prevalence of Alcohol Use Disorder (AUD), there are limited therapeutic options to prevent drug relapse. A characteristic of AUD is the presence of negative affect during withdrawal, which increases the likelihood of relapse (Becker, 2008; Koob, 2021). Moreover, stressful events occurring during withdrawal can also increase negative affect and decrease the likelihood of sobriety (Becker, 2008; Koob, 2021; Sinha, 2001). Thus, understanding the pharmacology driving negative affect and stress can help improve outcomes during abstinence in individuals with AUD. Neuropeptide systems are drug target candidates for treating AUD due to their potential role in negative affect- and stress-related relapse. The neuropeptide Calcitonin Gene-Related Peptide (CGRP) has established roles in stress, fear, pain, and anxiety contexts (Hashikawa-Hobara et al., 2015; Hashikawa-et al., 2024; Jaramillo et al., 2021; Palmiter, 2018; Schorscher-Petcu et al., 2009; Viero et al., 2022). Monoclonal antibodies and antagonists targeting the CGRP system are shown to be safe and an effective treatment for migraines in humans (e.g., epitinezumab, erenumab; (Wang et al., 2021)) and are currently being considered for the treatment of hangover migraines (Hakim, 2025; Narouze, 2025). Given CGRP monoclonal antibodies and antagonists are well-tolerated and bioavailable in humans, inhibiting CGRP is a promising neuropeptide target for an AUD pharmacotherapy.

The role of CGRP neurons in modulating anxiety during alcohol withdrawal is unknown. Based on preclinical studies, CGRP is anxiogenic in alcohol-naïve conditions. Specifically, blocking CGRP signaling peripherally or intracerebroventricularly (ICV) alters stress responses and decreases anxiety-like behavior (Hashikawa-Hobara et al., 2015; Hashikawa-et al., 2024; Schorscher-Petcu et al., 2009; Viero et al., 2022). The anxiogenic effects of CGRP are believed to be in part modulated by CGRP-expressing neurons in the parabrachial nucleus (PBN [CGRP]), as their activation increased threat responses such as fear- and anxiety-like behavior in both sexes (Jaramillo et al., 2021; Palmiter, 2018; Bowen et al., 2020; Han et al., 2015; Jaramillo et al., 2020; Pyeon et al., 2025). PBN(CGRP) neurons send projections to the bed nucleus of the stria terminalis (BNST) (Jaramillo et al., 2021; Palmiter, 2018; Sarhan et al., 2005; van Rossum et al., 1997; Ye and Veinante, 2019). The BNST is a critical node for anxiety and stress, including negative affective states in alcohol-withdrawal (Koob, 2021; Calhoon and Tye, 2015). Thus, the projections to the BNST, position PBN(CGRP) neurons as a potential modulator of negative affect in naïve and alcohol-exposed conditions. Indeed, studies demonstrate that manipulating CGRP in the BNST or directly manipulating the PBN(CGRP)→BNST circuit in alcohol-naïve conditions modulates anxiety-like behavior (Jaramillo et al., 2020; Sink et al., 2011; Sink et al., 2013a; Sink et al., 2013b). Moreover, we have demonstrated that PBN(CGRP) neurons drive BNST activity that synchronized to anxiety-like behavior (Jaramillo et al., 2020), thereby demonstrating direct recruitment of the circuit during an anxiogenic state. Interestingly, circuit-specific modulation reveals a sex-specific role for the PBN→BNST circuit in anxiety, as activating BNST neurons that are innervated by PBN projections (BNST^PBN^) selectively increased anxiety-like behavior in females (Jaramillo et al., 2020). The PBN(CGRP)→BNST circuit is also sensitive to anxiety-like behavior induced by stress, as neurotransmission driven by PBN(CGRP) projections in the BNST is dysregulated weeks after a stress-induced anxiety-like state in males (Kayat et al., 2025). Thus, manipulating PBN(CGRP) neurons during stress may sex-specifically prevent stress-induced anxiety. The role of PBN(CGRP) neurons in modulating anxiety-like behavior or stress after alcohol exposure is unknown. Therefore, the goal of this study is to investigate if manipulating PBN(CGRP) neurons is anxiolytic during early withdrawal and protective against stress-induced anxiety in protracted withdrawal in both sexes.

Although limited, the current data suggests a relationship between CGRP and alcohol. A clinical study in patients with psoriasis demonstrates a polymorphism in the *Calca* gene, which encodes for CGRP, was associated with non-drinking patients compared to patients with a history of drinking (Guo et al., 2015). Preclinical studies also suggest a genetic predisposition to alcohol that is associated with the CGRP system, as in alcohol-naïve conditions alcohol-preferring rats demonstrated differential CGRP receptor and peptide expression in select brain regions (Hwang et al., 1995; Rossetti et al., 2019). Although it is unknown if at baseline PBN(CGRP) or BNST^PBN^ neurons are sex-specifically different, studies implicate that PBN(CGRP) activity is effected after alcohol exposure. Specifically, in males CGRP terminal-expression in the BNST changed with voluntary alcohol exposure in alcohol-preferring rats in a sub-region specific manner (Ehlers et al., 1999). Additionally, like stress, alcohol induces long-term changes in PBN(CGRP) circuit activity, as activating the BNST in adult female mice with a history of adolescent alcohol vapor exposure increased activity in PBN(CGRP) neurons (Albrechet-Souza et al., 2024). Given exposure to alcohol induces changes in PBN(CGRP) activity, and in alcohol-naïve conditions activating PBN(CGRP) is anxiogenic, we hypothesize that activating PBN(CGRP) and BNST^PBN^ neurons will drive anxiety-like responses in alcohol-naïve mice and inhibiting PBN(CGRP) in early alcohol withdrawal will decrease anxiety-like behavior. Additionally, we hypothesize that inhibiting PBN(CGRP) activity during stress exposure in protracted withdrawal will decrease stress-associated anxiety-like behaviors. We expect to find sex-differences in PBN(CGRP)-modulated behavior and quantity of CGRP + cells in the PBN and CGRP + innervated cells in the BNST. Overall, we expect our findings to demonstrate a role for PBN(CGRP) neurons in anxiety during early and protracted alcohol withdrawal to thereby begin to elucidate if inhibiting CGRP is a viable treatment for alleviating stress and negative affect in AUD.

## 2 Materials and methods

### 2.1 Animals

Female and male *Calca* ^CRE^ mice (B6.Cg-Calcatm1.1 [cre/EGFP]Rpa/J) were used for Experiments 1, 4 and 5. *Calca* ^CRE^ mice are a heterozygous genetic knock-in mouse model that express CRE-recombinase at the *Calca* locus with a C57BL/6J background (Carter et al., 2013). For Experiment 2, female and male C57BL/6J mice were used. For Experiment 3, female and male CGRP-DTR^GFP^ mice (Calcitonin gene-related peptide [cre/EGFP] (CGRPalpha DTR) were used. CGRP-DTR^GFP^ knock-in mice express the axonal tracer (farnesylated enhanced green fluorescent protein; GFP) and LoxP-stopped cell ablation construct (human diphtheria toxin receptor; hDTR) to the *Calca* locus (McCoy et al., 2013). CGRP-DTR^GFP^ mice used in this study did not undergo cell ablation via the hDTR and were instead used as is. Both transgenic lines were bred in-house and genotyped with Transnetyx (Transetyx, Inc, Cordova, TN, United States).

All mice were 10–12 weeks old at the start of experiments. Mice were single- (Experiment 1) or group-housed (Experiments 2-5) with water and food available *ad lib* in the home cage. The colony room was maintained on a 12-h light/dark cycle (lights on at 06:00) under controlled temperature (20 °C–25 °C) and humidity (30%–50%) levels. All experiments were conducted during the light phase, with the exception of CIE, which was conducted in the dark phase. Animals were under continuous care and monitoring by veterinary staff from the Vanderbilt Division of Animal Care or University of Kentucky Division of Lab Animal Resources. All procedures were carried out in accordance with the NIH Guide to Care and Use of Laboratory Animals and institutional guidelines and approved by the Institutional Animal Care and Use Committee at Vanderbilt University and the University of Kentucky.

### 2.2 Stereotaxic viral surgeries

For Experiments 1-2, 4 and 5, adult mice were anesthetized with isoflurane (Covetrus, Portland, ME, United States; initial dose = 3%; maintenance dose = 1.5%) for surgery performed using a stereotax (Leica Biosystems, Nussloch, Germany or RWD Life Science, Shenzhen, Guangdong, PR, China). Coordinates from bregma were used to target the PBN (AP = −5.34, ML±1.31, DV = −3.37, 15.03° angle). Additionally, for Experiment 2 the BNST was targeted (AP = 0.14, ML ± 0.88, DV = −4.18, 15.03° angle). Care was taken to prevent drying of the eye by applying artificial tears ocular lubricant (Akorn, Lake Forest, IL, United States) and reapplying as needed throughout the surgery. Viral vectors were infused bilaterally at 300 nL (40 nL/min) with the needle remaining in place for an additional 5-min before withdrawal. Experiment 1 mice received a CRE-dependent virus expressing excitatory DREADDs (AAV5-hSyn-DIO-hM3D [Gq]-mCherry, hM3D [Gq]; titer ≥7.0 × 10^12^ vg/mL, Addgene, Watertown, MA, USA) in the PBN. Experiment 2 mice received a virus expressing CRE (AAV1-hSyn-CRE; titer ≥3.3 × 10^13^ vg/mL, Vigene, Rockville, MD, USA) in the PBN and a CRE-dependent virus expressing excitatory DREADDs (AAV5-hSyn-DIO-hM3D [Gq]-mCherry, hM3D [Gq]; titer ≥7.0 × 10^12^ vg/mL, Addgene, Watertown, MA, USA) in the BNST. For Experiments 4 and 5, mice received a CRE-dependent virus expressing inhibitory DREADDs (AAV5-hSyn-DIO-hM4D [Gi]-mCherry, hM4D [Gi]; titer ≥7 × 10^12^ vg/mL, Addgene, Watertown, MA, USA). Mice were given 0.9% saline for fluid maintenance postoperatively and body weights were tracked daily to ensure body weight was maintained. Mice were treated with Alloxate (Pivetal, Liberty, MO, United States; 2.5 mg/kg, S.C.) or Meloxicam (Covetrus, Portland, Maine, United States; 2.5 mg/kg S.C.) for 72-h after surgery and allowed to recover for 1 week. To allow for sufficient DREADD expression, mice received chemogenetic manipulations after >4 weeks. Viral deposits were confirmed post-testing utilizing autofluorescence or immunofluorescence protocols described below.

### 2.3 Immunofluorescence and viral vector confirmation

For all experiments, mice were deeply anesthetized using isoflurane, transcardially perfused with ice-cold phosphate buffered saline (PBS) followed by 4% paraformaldehyde (PFA) in PBS. Brains were submerged in 4% PFA for 24-h at 4 °C and cryoprotected in 30% sucrose in PBS for a minimum of 5 days. Coronal sections were cut on a cryostat (Leica CM3050S, Leica Biosystems, Nussloch, Germany) in Optimal Cutting Temperature solution (VWR, Radnor, PA, United States) at a thickness of 40 μm and stored in PBS at 4 °C until immunofluorescence or autofluorescence analysis.

To visualize CGRP expression for Experiment 3, sections were incubated in goat anti-CGRP primary antibody (1:400; AB36001, Abcam, Waltham, MA, USA) for 48-h at room temperature, washed in PBS, and incubated in Cy3 donkey anti-goat secondary antibody (1:500; Cy3 AffiniPure Donkey Anti-Goat, 705–165-003, Jackson, West Grove, PA, USA) in 0.1% Triton X-100 in PBS for 24-h at 4 °C and incubated in DAPI (1:10,000; 17,509, AAT Bioquest, Sunnyvale, CA, USA) in de-ionized water. Sections were washed with PBS and mounted on Fisher Plus slides (Fisher Scientific, Waltham, MA, USA) and cover slipped with Poly AquaMount (Polysciences, Warrington, PA, USA) when dry. Images were taken on Nikon Multi Excitation TIRF microscope (Nikon Metrology Inc, Tokyo, Japan) at 20× magnification. FIJI software (NIH, Bethesda, MD, USA) was used to assess CGRP + immunofluorescence. First, cell bodies were identified by DAPI + immunofluorescence. In the PBN, the total number of DAPI + cells that co-expressed CGRP + immunofluorescence within the cell body were quantified. In the BNST the total number of DAPI + cells that were overlaid with CGRP + immunofluorescence within or surrounding the cell body were quantified in the BNST.

For viral placement confirmation, in Experiment 1, hM3D (Gq) expression was confirmed by imaging mCherry autofluorescence in the PBN with a Keyence BZ-X710 microscope (Keyence, Itasca, IL, United States) at 20× magnification. For Experiment 2, hM3D (Gq) expression was confirmed by imaging mCherry autofluorescence in the BNST with Nikon Multi Excitation TIRF microscope (Nikon Metrology Inc, Tokyo, Japan) at 20× magnification. Additionally, FIJI software (NIH, Bethesda, MD, USA) was used to assess the total quantity of BNST cells with hM3D (Gq) expression. Cell bodies were identified with DAPI + immunofluorescence and the total number of DAPI + cells that co-expressed mCherry + autofluorescence within the cell body were quantified. For Experiment 4-5, hM4D (Gi) expression was confirmed by imaging mCherry autofluorescence in the PBN with ECHO Revolve microscope (ECHO, San Diego, CA, United States) at 20× magnification.

### 2.4 Chronic intermittent ethanol (CIE)

Mice underwent CIE described in (Kayat et al., 2025; Becker and Hale, 1993). Briefly, daily CIE exposure was as follows: at 16:00 h mice received a combined intraperitoneal (IP) injection of pyrazole (1 mmol/kg) + 1.6 g/kg ethanol and were immediately placed in an ethanol vapor (95% ethanol at 5 L/min) enclosure (VIPER system, LJari, La Jolla, CA, United States) until 08:00 h (total 16 h per day). Next, mice were returned to standard animal housing for 8 h (08:00–16:00 h). Mice were exposed to 2 CIE cycles consisting of: IP injection + vapor over the course of 4 days, followed by a 3-day stay in regular animal housing, and then another 4 days of IP injection + vapor. Mice did not receive any drug manipulation or behavior test during the CIE exposure.

### 2.5 Behavioral testing

For all testing, mice were brought into the testing room and allowed to acclimate for 1 hour. Mice were visualized, recorded, and tracked by an overhead camera using AnyMaze software (Stoelting Co, Wood Dale, IL, United States). Behavioral analyses were conducted by an observer blinded to treatment conditions.

#### 2.5.1 Elevated plus maze (EPM)

The maze is elevated 55 cm above the ground and consists of two open arms and two closed arms (30.5 × 6.5 cm; 16 cm closed arm height) with a 5 × 5 cm open center zone. Overhead lighting was placed above the apparatus such that light was limited to the open arms and did not create shadows. Lux was measured at the center of each open arm at 85–105 lux, center at 70 lux, and each closed arm at 0–6 lux by using a LX1010B Digital Light Meter (FTVOGUE, China). Mice were placed in the center facing alternate arms and recorded for 5 min.

#### 2.5.2 Forced swim stress (FSS)

FSS was performed as previously described (Kayat et al., 2025). Briefly, mice were placed into a transparent cylindrical container (i.e., 5000 mL glass beaker) with water at room temperature (20.6°–21.1 °C) for 6 min. Lux was measured at the center of the surface of the water at 300–350 lux using a LX1010B Digital Light Meter (FTVOGUE, China). Water was changed in between animals. The last 4 min of the test were analyzed for immobility.

#### 2.5.3 Novelty-suppressed feeding test (NSFT)

The 3-day NSFT was performed as previously described (Jaramillo et al., 2020; Kayat et al., 2025; Samuels and Hen, 2011). Briefly, mice were food restricted for 48 h except for 2 h food access 22–24 h before testing. The testing apparatus was a 50 × 50 cm arena with fresh home cage bedding and a single chow pellet at the center. Lux was measured at the center of the arena at 300–450 lux with a LX1010B Digital Light Meter (FTVOGUE, China). At the start of testing, mice were placed in a corner of the arena. Food approach frequency was defined as approach to the 10 cm diameter around the center-located food pellet. Mice were removed from the testing arena immediately after the first bite (i.e., consummatory food approach) and placed in their home cage with the food pellet from the test. The pellet was weighed after 10 min to confirm food consumption. Mice were returned to *ad lib* access to chow post testing. Latency to the first bite and food consumption were hand-scored by an observer blinded to treatment groups.

### 2.6 Experimental procedures

#### 2.6.1 Experiment 1: effects of PBN(CGRP) activation on EPM

**FIGURE 1:**
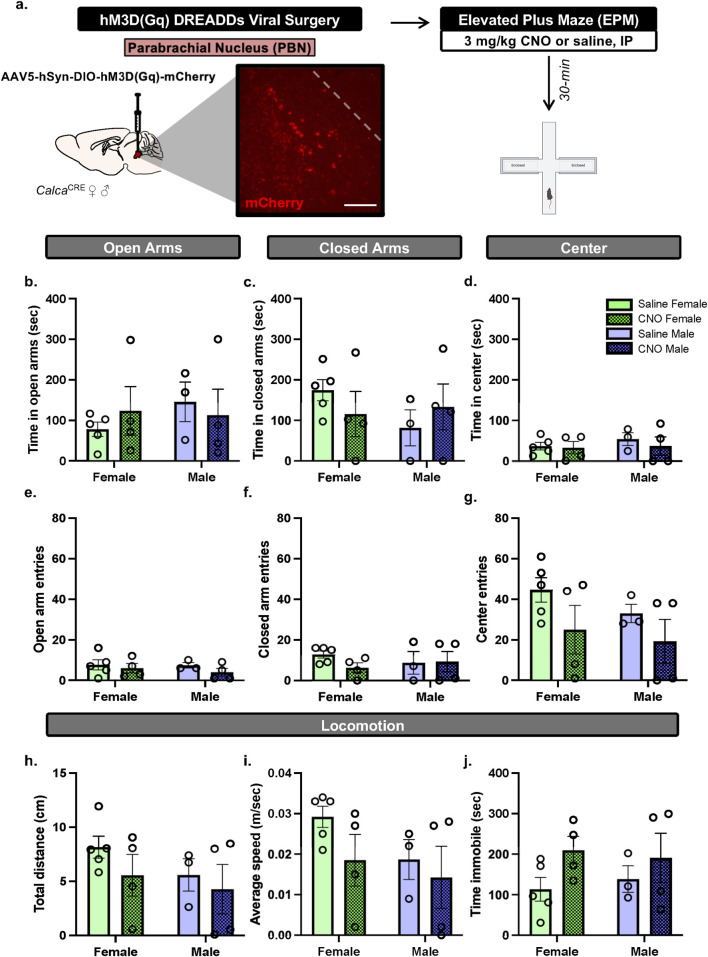
PBN(CGRP) activation does not change EPM behaviors in either sex **(a)** *Calca* ^Cre^ female and male mice received bilateral infusions to express CRE-dependent hM3D (Gq) DREADDs in the PBN and were tested with EPM post-30 min pretreatment of CNO (3 mg/kg, IP). Image (200 μM scale bar) demonstrates representative hM3D (Gq) (mCherry) autofluorescence in the PBN. **(b)** CNO did not change time in open arms, **(c)** time in closed arms, **(d)** time in center, **(e)** open arm entries, **(f)** closed arm entries, **(g)** center entries, **(h)** distance travelled, **(i)** average speed or **(j)** time immobile in EPM compared to saline. Values on graphs represent mean ± S.E.M. (Two-way ANOVA, Tukey’s; *p≦0.05).

First, to determine the role of the PBN(CGRP) and BNST^PBN^ neurons on anxiety-like behavior in alcohol-naïve conditions, we focus on behavior during EPM. *Calca* ^CRE^ female (n = 9) and male mice (n = 7) received bilateral injections of CRE-dependent virus to express hM3D (Gq) in the PBN, as previously reported in (15). Mice with inefficient viral expression (2 females) received saline treatment and therefore were included in the data. Mice received Clozapine-N-oxide (CNO, 3 mg/kg, intraperitoneal, I.P.) or saline in their home cage 30-min prior to the start of EPM testing (Figure 1).

#### 2.6.2 Experiment 2: effects of BNST^PBN^ activation on EPM

**FIGURE 2:**
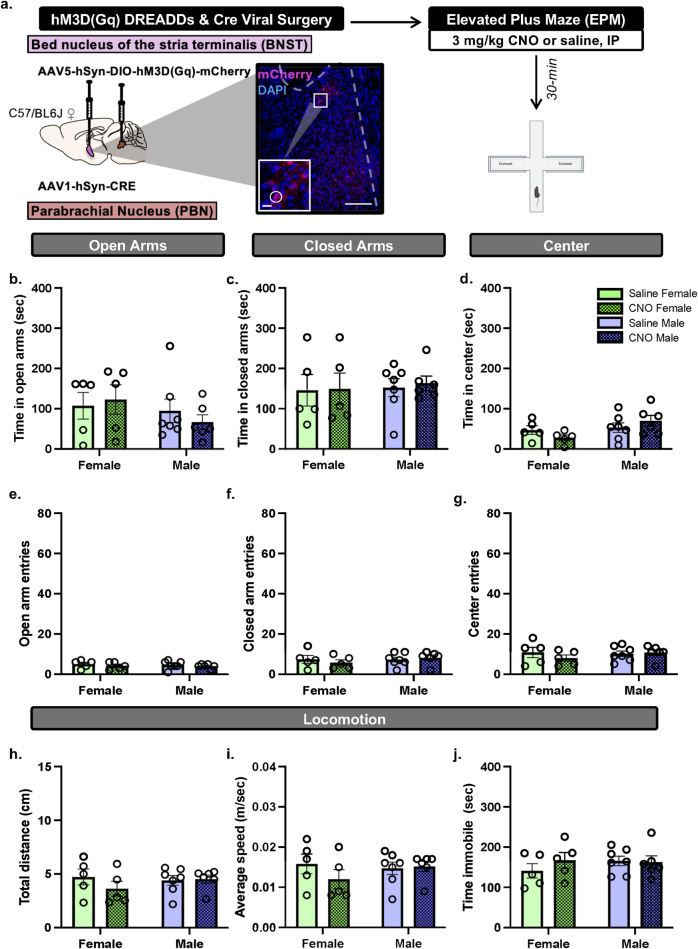
BNST^PBN^ activation does not change EPM behaviors in either sex **(a)** *Calca* ^Cre^ female and male mice received bilateral infusions of AAV1-CRE anterograde transsynaptic virus in the PBN and CRE-dependent hM3D (Gq) DREADDs virus in the BNST, to express hM3D (Gq) DREADDs in BNST neurons that are innervated by PBN projections. Mice were tested with EPM post-30 min pretreatment of CNO (3 mg/kg, IP). Image (200 μM scale bar) and inset (67 μM scale bar) demonstrates representative hM3D (Gq) (mCherry) autofluorescence in the BNST. **(b)** CNO did not change time in open arms, **(c)** time in closed arms. **(d)** nor time in center. **(e)** CNO did not change open arm entries, **(f)** closed arm entries, **(g)** center entries, **(h)** distance travelled, **(i)** average speed, nor **(j)** time immobile in EPM compared to saline. Values on graphs represent mean ± S.E.M. (Two-way RM ANOVA, Tukey’s; *p≦0.05).

To determine the role of the BNST^PBN^ neurons on anxiety-like behavior in alcohol-naïve conditions, we focus on behavior during EPM. C57BL/6J female (n = 8) and male mice (n = 7) received bilateral injections of the transsynaptic anterograde CRE-expressing virus in the PBN and CRE-dependent virus in the BNST. Thereby, hM3D (Gq) would be expressed in BNST neurons that receive projections from the PBN, as previously reported in (Jaramillo et al., 2020). Mice with inefficient viral expression (3 females) were not included in the data. Mice received CNO (3 mg/kg, intraperitoneal, I.P.) or saline in their home cage 30-min prior to the start of EPM testing (Figure 2). Using a within-subject design, mice were re-tested with EPM albeit with the opposite treatment (e.g., mice that received CNO for the first EPM test now received saline). Repeated daily or weekly testing with EPM can induce one-trial tolerance (Tucker and McCabe, 2017) albeit not in all cases (Lister, 1987; Pellow et al., 1985; Schneider et al., 2011). Therefore, the second EPM test occurred 3 weeks from the initial EPM test, as this time window between testing has been shown in part to avoid behavioral tolerance (Schneider et al., 2011).

#### 2.6.3 Experiment 3: CGRP expression in PBN neurons and fibers in the BNST

**FIGURE 3:**
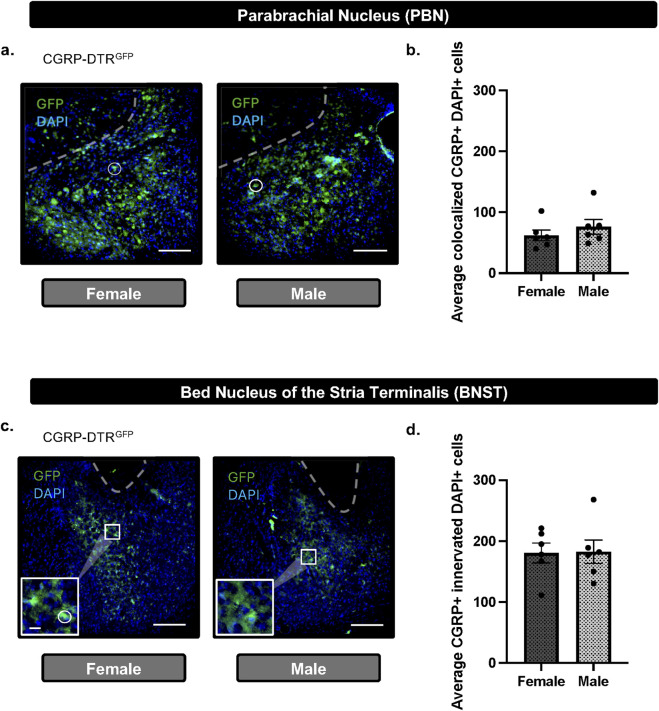
CGRP + cells in the PBN and CGRP + innervated cells in the BNST do not differ between females and males **(a)** Representative image (scale bar 200 μM) and DAPI merged (white circle) immunofluorescence in the PBN of CGRP-DTR^GFP^ females and males. **(b)** Quantification of CGRP (GFP) demonstrate no difference in total CGRP + DAPI colocalization in the PBN between males and females. **(c)** Representative image (200 μM scale bar) and inset (67 μM scale bar) demonstrating immunofluorescence of CGRP (GFP) and DAPI merged (white circle) in the BNST of CGRP-DTR^GFP^ females and males. **(d)** Quantification of CGRP (GFP) demonstrate no difference in total CGRP-innervated DAPI + colocalization in the PBN between males and females. Values on graphs represent mean ± S.E.M. (unpaired *t*-test; *p≦0.05).

To determine if there are sex-differences in PBN(CGRP) neurons and CGRP-innervated BNST neurons, we quantified CGRP neuron expression in the PBN and fiber expression in the BNST. CGRP-DTR^GFP^ female (n = 6) and male (n = 6) mice were behaviorally-naïve (i.e., no behavioral assay exposure) and experimentally-naïve (i.e., no cell ablation). PBN-containing slices were analyzed for co-localization of CGRP (GFP) and DAPI (i.e., within the cell body) and reported as total co-localized (Figures 3a,b). BNST-containing slices were analyzed for co-localization of DAPI innervated by CGRP (GFP) expression (i.e., within or surrounding the cell body) and reported as total co-localized (Figures 3c,d).

#### 2.6.4 Experiment 4: effects of PBN(CGRP) inactivation on EPM during acute CIE withdrawal

**FIGURE 4:**
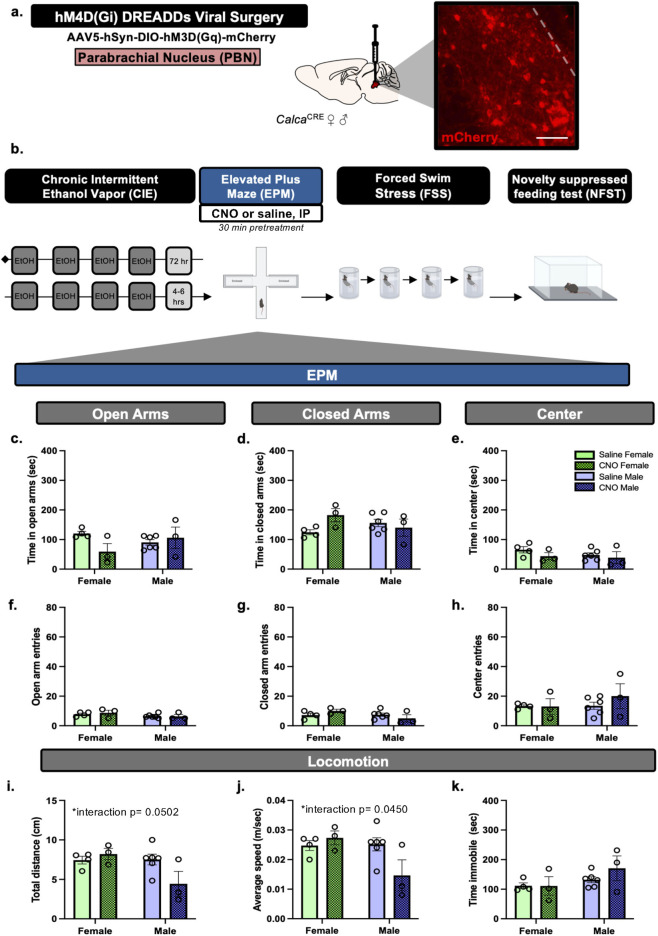
PBN(CGRP) inactivation during acute withdrawal from CIE sex-specifically decreases locomotor behaviors in EPM **(a)** *Calca* ^Cre^ female and male mice received bilateral viral infusions to express CRE-dependent hM4D (Gi) DREADDs in the PBN. Inset demonstrates representative image (200 μM scale bar) of hM4D (Gi) (mCherry) autofluorescence in the PBN. **(b)** Mice received two cycles of CIE vapor exposure (one cycle = 4 days of 16 h of vapor, depicted in dark gray) with a 72 h withdrawal between cycles. Next, during acute withdrawal (4–6 h post-final CIE exposure), mice were tested with EPM post-30 min pretreatment of CNO (3 mg/kg, IP). **(c)** CNO did not change time in open arms, **(d)** time in closed arms, **(e)** time in center, **(f)** open arm entries, **(g)** closed arm entries, nor **(h)** center entries in EPM compared to saline. **(i)** CNO sex-specifically decreased distance travelled (p = 0.0502), and **(j)** average speed (p = 0.0450). **(k)** CNO did not change time immobile compared to saline. Values on graphs represent mean ± S.E.M., (Two-way ANOVA, Tukey’s; *p≦0.05).

Next, we utilized chronic intermittent ethanol vapor exposure (CIE) and EPM to determine the impact of PBN(CGRP) inhibition on anxiety-like behavior in acute withdrawal. *Calca* ^CRE^ female (n = 7) and male (n = 10) mice received CRE-dependent viral injection to express hM4D (Gi) in the PBN. Mice with inefficient viral expression (2 female, 6 males) were included in the data as appropriate (i.e., received saline treatment). Mice were exposed to two cycles of CIE vapor. During acute withdrawal from CIE (4–6 h) mice received CNO (3 mg/kg, I.P.) or saline in their home cage 30-min prior to the start of EPM testing (Figure 4).

#### 2.6.5 Experiment 5: effects of PBN(CGRP) inactivation on NSFT following repeated FSS during protracted CIE withdrawal

**FIGURE 5:**
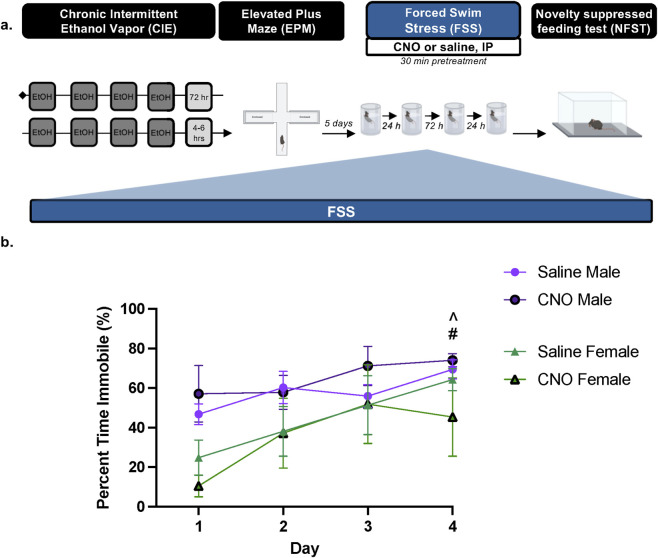
PBN(CGRP) inactivation in females disrupts increased FSS immobility across time in protracted withdrawal from CIE **(a)** 1 week post CIE (i.e., 1 week after EPM), *Calca* ^Cre^ female and male mice expressing CRE-dependent hM4D (Gi) DREADDs in the PBN received CNO (3 mg/kg, IP) or saline 30 min prior to a 6 min forced swim stress (FSS) for 4 days **(b)** In saline-treated females, percent time immobile increased on Day 4 relative to Day 1 (^#^p = 0.0072). Saline-treated males had a higher percent time immobile on Day 4 relative to saline-treated females on Day 1 (^^^ p = 0.0141). Values on graphs represent mean ± S.E.M. (Three-way RM ANOVA, Bonferroni’s; p≦0.05).

**FIGURE 6:**
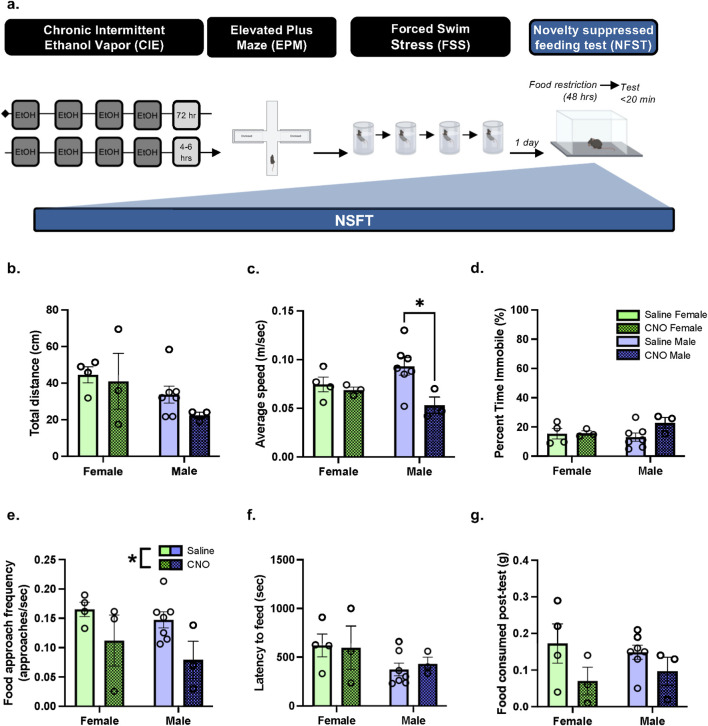
In protracted withdrawal from CIE, PBN(CGRP) inactivation during FSS decreased subsequent approach frequency in both sexes and decreased speed in males in NSFT **(a)** 2 weeks post CIE*, Calca* ^Cre^ female and male mice with a history of CNO or saline treatment during FSS, were tested with NSFT. No chemogenetic manipulations occurred during NSFT. **(b)** Distance travelled was similar with a history of Saline + FSS and CNO + FSS. **(c)** In males, a history of CNO + FSS decreased average speed relative to saline treatment (p = 0.0369) **(d)** Percent time immobile was similar with a history of Saline + FSS and CNO + FSS. **(e)** A history of CNO + FSS decreased approach frequency relative to Saline + FSS (p = 0.0227). **(f)** Latency to feed **(g)** and food consumed post-test were similar with a history of Saline + FSS and CNO + FSS. Values on graphs represent mean ± S.E.M. (Two-way ANOVA, Tukey’s; *p≦0.05).

To evaluate the anxiolytic potential of repeated PBN(CGRP) inhibition during stress in protracted withdrawal from CIE, we utilized repeated forced swim stress (FSS) and novelty suppressed feeding task (NSFT), two paradigms in which behavior has been shown to be modulated by CGRP or PBN(CGRP) neurons (Hashikawa-Hobara et al., 2015; Jaramillo et al., 2020; Kayat et al., 2025). 1 week into withdrawal from CIE, *Calca* ^CRE^ female and male mice from Experiment 4 underwent FSS, previously described in (Kayat et al., 2025). Mice were exposed as follows: 2 days of FSS, 2-day incubation period (i.e., no FSS), and 2 days of FSS. Mice received CNO (3 mg/kg, I.P.) or saline in their home cage 30-min prior to the each FSS (Figure 5). Treatment assignments remained consistent with Experiment 4. After the final FSS, all mice underwent the 3-day NSFT paradigm (Figure 6). No CNO or saline treatment was given prior to NSFT.

### 2.7 Drugs

Sterile saline or 3 mg/kg Clozapine-N-oxide (CNO, MilliporeSigma, diluted in sterile saline) was delivered I.P. Pyrazole + ethanol (1 mmol/kg Pyrazole + 1.6 g/kg ethanol) diluted in sterile saline was delivered I.P.

### 2.8 Statistical analysis

Data is represented as mean ± S.E.M and all statistics were run using Prism 10 (GraphPad). Differences between groups were assessed using ordinary and Repeated Measures (RM) Two-way analysis of variance (ANOVA) with Tukey’s *post hoc* tests, and Three-way RM ANOVA with Bonferroni’s *post hoc* tests. Significant ANOVA main effects/interactions and *post hoc* tests were followed up with Cohen’s Partial Eta Squared (η^2^_p_) and Cohen’s *d* tests, respectively, to determine effect size. Significance was set at p ≦ 0.05.

## 3 Results

### 3.1 Experiment 1: PBN(CGRP) activation does not alter behaviors in EPM

To assess whether activating PBN(CGRP) neurons modulates behavior in EPM, *Calca* ^CRE^ female and male mice received bilateral viral injections of CRE-dependent hM3D (Gq) DREADDs virus in the PBN (Figure 1a). CNO (3 mg/kg, IP) or vehicle (saline) treatment was administered 30-min prior to EPM. Two-way ANOVA of total time spent in EPM zones demonstrates no significant effect on time in open arms (Figure 1b; Treatment: F [1,12] = 0.01624, p = 0.9007; Sex: F [1,12] = 0.3340, p = 0.5740; Treatment × Sex F [1,12] = 0.6244, p = 0.4447), closed arms (Figure 1c; Treatment: F [1,12] = 0.005830, p = 0.9404; Sex: F [1,12] = 0.6523, p = 0.4350; Treatment × Sex: F [1,12] = 1.416, p = 0.2571), nor center zone (Figure 1d; Treatment: F [1,12] = 0.4342, p = 0.5224; Sex: F [1,12] = 0.4326, p = 0.5231; Treatment × Sex: F [1,12] = 0.1716, p = 0.6860). Therefore, hM3D (Gq) activation by CNO did not affect time spent in the EPM areas. Two-way ANOVA of number of entries into zones of the EPM demonstrates no significant effect on number of entries in open arms of the EPM (Figure 1e; Treatment F [1,12] = 1.178, p = 0.2990; Sex F [1,12] = 0.2487, p = 6270; Treatment × Sex interaction F [1,12] = 0.1455, p = 0.7096), closed arms (Figure 1f; Treatment F [1,12] = 0.6615, p = 0.4319; Sex (F [1,12] = 0.02387; p = 0.8798; Treatment × Sex interaction (F [1,12] = 0.9455, p = 0.3501), nor center zone (Figure 1g; Treatment F [1,12] = 3.252, p = 0.0965; Sex (F [1,12] = 0.8801, p = 0.3667; Treatment × Sex interaction (F [1,12] = 0.1001, p = 0.7572). Therefore, hM3D (Gq) activation by CNO did not affect number of entries into the EPM areas. Two-way ANOVA of locomotor behaviors in the EPM demonstrates no significant effect on total distance travelled (Figure 1h; Treatment (F [1,12] = 1.255, p = 0.2846; Sex F [1,12] = 1.213, p = 0.2923; Treatment × Sex interaction F [1,12] = 0.1351, p = 0.7196), average speed (Figure 1i; Treatment F [1,12] = 1.799, p = 0.2047; Sex (F [1,12] = 1.720, p = 0.2142; Treatment × Sex interaction F [1,12] = 0.3108, p = 0.5874), nor total time immobile (Figure 1j; Treatment F [1,12] = 3.096, p = 0.1039; Sex F [1,12] = 0.005192, p = 0.9437; Treatment × Sex interaction F [1,12] = 0.2765, p = 0.6086). Therefore, hM3D (Gq) activation by CNO did not affect locomotor behaviors in the EPM. Overall, in both sexes, activating excitatory hM3D (Gq) DREADDs expressed in the PBN via CNO did not change behaviors in EPM.

### 3.2 Experiment 2: BNST^PBN^ activation does not alter behaviors in EPM

To assess whether activating of BNST^PBN^ neurons modulates behavior in EPM, we used an anterograde transsynaptic viral transfer strategy (Zingg et al., 2017; Centanni et al., 2019; Zingg et al., 2020) that would target PBN-innervated BNST cells. C57BL/6J female and male mice received bilateral injections of anterograde transsynaptic CRE virus in the PBN and CRE-dependent hM3D (Gq) DREADDs virus in the BNST (Figure 2a) which resulted in average total 140.3 ± 20.28 (female) and 111.5 ± 29.45 (male) BNST cells expressing hM3D (Gq). CNO (3 mg/kg, IP) or vehicle (saline) treatment was administered 30-min prior to EPM. Two-way ANOVA of total time spent in EPM zones demonstrates no significant effect on time in open arms (Figure 2b; Treatment (F [1,19] = 0.04550, p = 0.8334; Sex (F [1,19] = 1.334, p = 0.2623; Treatment × Sex interaction F [1,19] = 0.5331, p = 0.4742) nor closed arms (Figure 2c; Treatment F [1,19] = 0.06181, p = 0.8063; Sex F [1,19] = 0.1236, p = 0.7290; Treatment × Sex interaction F [1,19] = 0.01638, p = 0.8995). Two-way ANOVA of total time spent in the center zone of the EPM demonstrates a trend towards a significant main effect of Sex (Figure 2d; F [1,19] = 4.181, p = 0.0550), and no significant main effect of Treatment (F [1,19] = 0.006770, p = 0.9353), nor Treatment × Sex interaction (F [1,19] = 2.337, p = 0.1428). Therefore, hM3D (Gq) activation by CNO did not affect time spent in the EPM areas. Two-way ANOVA of number of entries into the arms of the EPM demonstrates no significant effect on entries in open arms (Figure 2e; Treatment F [1,19] = 0.8607, p = 0.3652; Sex (F [1,19] = 0.1808, p = 0.6755; Treatment × Sex interaction F [1,19] = 0.02391, p = 0.8787), closed arms (Figure 2f; Treatment F [1,19] = 0.06174, p = 0.8064; Sex F [1,19] = 0.6058, p = 0.4460; Treatment × Sex interaction F [1,19] = 0.7350, p = 0.4020), nor center zone (Figure 2g; Treatment F [1,19] = 0.4296, p = 0.5201; Sex F [1,19] = 0.3348, p = 0.5696; Treatment × Sex interaction F [1,19] = 0.9159, p = 0.3506). Therefore, hM3D (Gq) activation by CNO did not affect number of entries into the EPM areas. Two-way ANOVA of locomotor behaviors in the EPM demonstrates no significant effect on total distance travelled (Figure 2h; Treatment (F [1,19] = 0.7572, p = 0.3951; Sex F [1,19] = 0.2696, p = 0.6096; Treatment × Sex interaction F [1,19] = 1.171, p = 0.2926), average speed (Figure 2i; Treatment F [1,19] = 0.7875, p = 0.3859; Sex F [1,19] = 0.3043, p = 0.5876; Treatment × Sex interaction F [1,19] = 1.271, p = 0.2737), nor total time immobile (Figure 2j; Treatment F [1,19] = 0.4815, p = 0.4962; Sex (F [1,19] = 0.3718, p = 0.5493; Treatment × Sex interaction F [1,19] = 0.8441, p = 0.3697). One animal was a significant outlier based on open arm time (p < 0.05, z = 2.063) and speed (p < 0.05, z = 2.0303) and thus was omitted. Therefore, hM3D (Gq) activation by CNO did not affect locomotor behaviors in the EPM. Overall, in both sexes, activating excitatory hM3D (Gq) DREADDs, expressed in BNST^PBN^ neurons, via CNO did not change behaviors in EPM.

### 3.3 Experiment 3: CGRP + cell expression in the PBN and CGRP + innervated cells in the BNST do not differ between sexes

To assess whether CGRP-containing cells in the PBN and CGRP-innervated cells in the BNST differed between the sexes, the PBN (Figure 3a) and BNST (Figure 3c) CGRP-DTR^GFP^ female and male mice were analyzed for CGRP expression. Mice were behaviorally-naïve (i.e., no behavioral assay exposure) and experimentally-naïve (i.e., no cell ablation). Unpaired *t*-test of total colocalized CGRP (GFP) and DAPI cells in PBN slices demonstrated no significant difference in females relative to males (t_10_ = 0.9321, p = 0.3732; Figure 3b). Similarly, unpaired *t*-test of total colocalized CGRP (GFP) innervated DAPI cells in PBN slices demonstrated no significant difference in females relative to males (t_10_ = 0.07935, p = 0.9383; Figure 3d). Overall, the baseline quantity of CGRP-containing PBN cells or CGRP-innervated cells does not differ between females and males.

### 3.4 Experiment 4: PBN(CGRP) inactivation during acute alcohol withdrawal sex-specifically decreases locomotor behavior in EPM

To assess whether inactivating PBN(CGRP) neurons modulates EPM behavior during early withdrawal from CIE, *Calca* ^CRE^ female and male mice received bilateral injections of CRE-dependent hM4D (Gi) DREADDs virus in the PBN (Figure 4a). Mice underwent two cycles of CIE vapor exposure. During acute withdrawal (4–6 h post final CIE exposure), CNO (3 mg/kg, IP) or vehicle (saline) treatment was administered 30-min prior to EPM (Figure 4b). Two-way ANOVA of total time spent in open arms of the EPM (Figure 4c) demonstrates a trend towards a significant Treatment × Sex interaction (F [1,12] = 4.250, p = 0.0616), and no significant main effect of Treatment (F [1,12] = 1.469, p = 0.2488), nor Sex (F [1,12] = 0.2066, p = 0.6576). Two-way ANOVA of total time spent in closed arms of the EPM (Figure 4d) demonstrates a trend towards a significant Treatment × Sex interaction (F [1,12] = 4.434, p = 0.0570), and no significant main effect of Treatment (F [1,12] = 1.426, p = 0.2555), nor significant main effect of Sex (F [1,12] = 0.09428, p = 0.7641). Two-way ANOVA of total time spent in the center zone (Figure 4e) of the EPM demonstrates no significant main effect of Treatment (F [1,12] = 1.656, p = 0.2224), Sex (F [1,12] = 0.9067, p = 0.3598), nor Treatment × Sex interaction (F [1,12] = 0.2808, p = 0.6058). Therefore, hM3D (Gq) activation by CNO did not affect time spent in the EPM areas. Two-way ANOVA of number of entries into the arms of the EPM demonstrates no significant effect on entries in open arms (Figure 4f; Treatment (F [1,12] = 0.1309, p = 0.7237; Sex (F [1,12] = 2.989, p = 0.1094; Treatment × Sex interaction F [1,12] = 0.2732, p = 0.61070, closed arm (Figure 4g; Treatment (F [1,12] = 0.006906, p = 0.9351; Sex (F [1,12] = 2.493, p = 0.1403; Treatment × Sex interaction F [1,12] = 3.045, p = 0.1065), nor center zone (Figure 4h; Treatment F [1,12] = 0.5603, p = 0.4686; Sex F [1,12] = 0.7539, p = 0.4023; Treatment × Sex interaction F [1,12] = 0.6535, p = 0.4346). Therefore, hM3D (Gq) activation by CNO did not affect number of entries into the EPM areas. Two-way ANOVA of total distance travelled in the EPM (Figure 4i) demonstrates a significant Treatment × Sex interaction (F [1,12] = 4.736, p = 0.0502, η^2^_p_ = 0.7170), a trend towards a significant main effect of Sex (F [1,12] = 4.388, p = 0.0581), and no significant main effect of Treatment (F [1,12] = 1.711, p = 0.2154). Therefore, CNO sex-specifically affected distance travelled in EPM, with a trend towards decreased distance in males relative to females with CNO treatment (p = 0.0762). Two-way ANOVA of average speed in the EPM (Figure 4j) demonstrates a significant Treatment × Sex interaction (F [1,12] = 5.004, p = 0.0450, η^2^_p_ = 0.7057), a trend towards a significant main effect of Sex (F [1,12] = 4.387, p = 0.0581) and no significant main effect of Treatment (F [1,12] = 1.832, p = 0.2008). Therefore, CNO sex-specifically affected average speed in EPM, with a trend towards decreased speed in males relative to females with CNO treatment (p = 0.0714). Two-way ANOVA of total time immobile in the EPM (Figure 4k) demonstrate no significant main effect of Treatment (F [1,12] = 0.7810, p = 0.3942), Sex (F [1,12] = 3.465, p = 0.0873), nor Treatment × Sex interaction (F [1,12] = 0.8247, p = 0.3817). One animal was a significant outlier based on total time immobile (p < 0.05, z = 2.137) and close arm time (p < 0.05, z = 2.049) and thus was omitted. Overall, activating inhibitory hM3D (Gi) DREADDs expressed in the PBN via CNO sex-specifically decreased locomotor behaviors in EPM during acute withdrawal.

### 3.5 Experiment 5: PBN(CGRP) inactivation during FSS alters immobility in females and subsequent behavior in NSFT in both sexes

Next, we assessed whether inactivating PBN(CGRP) neurons during repeated FSS exposure modulates subsequent NSFT behavior during protracted withdrawal from CIE. On the sixth day of withdrawal from CIE, *Calca* ^CRE^ female and male mice from Experiment 4 were exposed to FSS for 4 days, with CNO (3 mg/kg, IP) or vehicle (saline) administered 30-min prior to testing (Figure 5a). Three days post-FSS, mice were tested on NSFT (Figure 6a). Three-way RM ANOVA of percent time immobile in FSS (Figure 5b) demonstrates a significant main effect of Day (F [3,39] = 10.88, p < 0.0001, η^2^_p_ = 0.9149), and main effect of Sex (F [1,13] = 6.719, p = 0.0223, η^2^_p_ = 0.9457), indicating in females immobility increased on day 4 relative to day 1 (p = 0.0072, d = 2.6463) and males had a higher immobility on day 4 relative to females on day 1 with saline treatment (p = 0.0141, d = 2.8723). There was no significant main effect of Treatment (F [1,13] = 0.007910, p = 0.9405), Day × Treatment interaction (F [3,39] = 0.6922; p = 0.5623), Day × Sex interaction (F [3,39] = 1.612, p = 0.2022), Treatment × Sex interaction (F [1,13] = 0.8852, p = 0.3639), nor Day × Treatment × Sex three-way interaction (F [3,39] = 0.6413, p = 0.5930). Overall, activating the inhibitory hM4D (Gi) DREADDs expressed in the PBN via CNO disrupted increased immobility across FSS exposure during protracted CIE-withdrawal in females.

In mice with history of FSS + CNO or FSS + Saline, two-way ANOVA of total distance travelled in NSFT (Figure 6b) demonstrates a trend towards a significant main effect of Sex (F [1,13] = 4.184, p = 0.0616), and no significant main effect of History (F [1,13] = 1.077, p = 0.3183), nor History × Sex interaction (F [1,13] = 0.2946, p = 0.5965). Two-way ANOVA of average speed in NSFT (Figure 6c) demonstrates a significant main effect of History (F [1,13] = 5.688, p = 0.0330, η^2^_p_ = 0.6956), and no significant main effect of Sex (F [1,13] = 0.02174, p = 0.8851), nor significant History × Sex interaction (F [1,13] = 3.161, p = 0.0988), indicating in males a history of CNO decreased speed relative to saline history (p = 0.0369, d = 2.0287). Two-way ANOVA of percent time immobile in NSFT (Figure 6d) demonstrates no significant main effect of History (F [1,13] = 2.202, p = 0.1617), Sex (F [1,13] = 0.4625, p = 0.5084), nor History × Sex interaction (F [1,13] = 1.836, p = 0.1985). Two-way ANOVA of approach frequency in NSFT (Figure 6e) demonstrates a significant main effect of History (F [1,13] = 6.681, p = 0.0227, η^2^_p_ = 0.6605), and no significant main effect of Sex (F [1,13] = 1.162, p = 0.3007), nor History × Sex interaction (F [1,13] = 0.1022, p = 0.7543), thereby a history of CNO decreased approach frequency. Two-way ANOVA of latency to feed in NSFT (Figure 6f) demonstrates no significant main effect of History (F [1,13] = 0.02122, p = 0.8864), Sex (F [1,13] = 3.143, p = 0.0997), nor History × Sex interaction (F [1,13] = 0.1203, p = 0.7343). Two-way ANOVA of amount of food consumed post-NSFT (Figure 6g) demonstrates a trend towards a significant main effect of History (F [1,13] = 4.313, p = 0.0582), and no significant main effect of Sex (F [1,13] = 0.001356, p = 0.9712), nor History × Sex interaction (F [1,13] = 0.4631, p = 0.5081). Overall, a history of activating the inhibitory hM4D (Gi) DREADDs expressed in the PBN via CNO paired with FSS decreased approach frequency in females and males and decreased speed travelled in males during NSFT in protracted CIE-withdrawal.

## 4 Discussion

In this study, we demonstrate chemogenetically activating PBN(CGRP) or BNST^PBN^ neurons during EPM did not change behaviors under alcohol-naïve conditions for either sex. Additionally, experimentally-naïve females and males showed no differences in number of CGRP + cells in the PBN and CGRP + innervated cells in the BNST. Conversely, during acute withdrawal from CIE inhibiting PBN(CGRP) neurons in EPM sex-specifically decreased locomotion. In the same cohort, inhibiting PBN(CGRP) neurons during repeated FSS in protracted withdrawal disrupted adaptative immobility behavior in females. Subsequent testing in NSFT demonstrated a history of inhibiting PBN(CGRP) neurons during FSS increased negative affect in both sexes, while selectively decreasing locomotion in males. Thus, our findings demonstrate that manipulating PBN(CGRP) neurons induced sex-specific changes in locomotion across alcohol-withdrawal and resulted in a negative affective state in both sexes.

Previous work in both sexes demonstrates activating PBN(CGRP) projections increases anxiety-like and avoidance behaviors in passive and active avoidance assays. Specifically, optogenetically activating PBN(CGRP) neurons increased freezing behavior and decreased open arm time and preference in EPM in both sexes (Bowen et al., 2020). Additionally, optogenetic stimulation of PBN(CGRP) projections also led to avoidance of the stimulation side of the real-time place-preference chamber (Bowen et al., 2020). Similar effects were found with circuit-specific optogenetic activation of PBN to paraventricular thalamus projections in male mice, as anxiety-like behaviors increased in the open field test and increased avoidance of the photostimulation-paired chamber in real-time place aversion (Zhu et al., 2022). Studies that pharmacologically manipulate CGRP also demonstrate an anxiogenic role, as intra-BNST infusions of CGRP agonist enhanced anxiety-like measures in EPM (Sink et al., 2011; Sink et al., 2013a). Given the PBN is the dominant extrinsic source of CGRP in the BNST (Jaramillo et al., 2021; Palmiter, 2018; Sarhan et al., 2005; van Rossum et al., 1997; Ye and Veinante, 2019), these studies indirectly implicate a role for the PBN(CGRP)→BNST circuit in modulating anxiety. However, our findings demonstrate chemogenetic activation of PBN(CGRP) neurons did not change anxiety-like behaviors during EPM in either sex. Moreover, circuit specific manipulations of BNST^PBN^ neurons only demonstrated a trend for sex-specific differences in time in center. The lack of effect with BNST^PBN^ manipulations may be due to the limited BNST^PBN^ neurons that expressed DREADDs in comparison to the quantity of CGRP innervated cells in the BNST that were noted in the CGRP-DTR^GFP^ mice. The modest expression may be due to limitations of the anterograde viral strategy (e.g., transfection), or due to the differences in endogenous quantity of BNST^PBN^ neurons in the 2 mouse strains (i.e., *Calca* ^CRE^ vs. CGRP-DTR^GFP^). Moreover, given the within-subject testing of BNST^PBN^ manipulations on EPM, it is possible that a one-trial tolerance occurred with repeated EPM testing (Tucker and McCabe, 2017), an effect that is in part avoided in the present study by the time in between testing (Schneider et al., 2011). Alternatively, the lack of effect induced by chemogenetically activating PBN(CGRP) and BNST^PBN^ neurons may be due to the mode of manipulations. The previous studies used optogenetic and pharmacological manipulations, which offer instant control of neuronal activity (Claes et al., 2022) and direct manipulation of an endogenous receptor, respectively. Given our use of DREADDs induces gradual neuromodulation through heighten excitability (Claes et al., 2022; Roth, 2016), the optogenetic and pharmacological manipulations during the passive and active avoidance assays may elicit significant anxiety-like responses due to their robust ability to engage neuronal control. Relative to other studies that used chemogenetic manipulations, our findings suggest that that the behavioral assay used to investigate the anxiogenic role of PBN(CGRP) neurons may also result in different effects. When measuring anxiety-like behavior with NSFT, an approach-avoidance conflict task, in females and males, chemogenetically activating PBN(CGRP) neurons increased latency to feed and frequency of non-consummatory approach bouts (Jaramillo et al., 2020). Circuit-specific chemogenetic BNST^PBN^ neuron activation also increased latency to feed albeit selectively in females (Jaramillo et al., 2020). Given the difference in behavioral assays, (i.e., EPM compared to NSFT), chemogenetically activating PBN and BNST^PBN^ neurons may be more sensitive to anxiety-like behavior in approach-avoidance contexts. Altogether, our studies suggest that in naïve conditions increasing PBN(CGRP) neuron excitability is not sufficient to induce anxiety-like behavior in a passive avoidance context.

While no change in EPM behaviors arose following chemogenetic PBN(CGRP) manipulation in alcohol-naïve mice, we demonstrate inhibiting PBN(CGRP) neurons sex-specifically affected locomotor behaviors in EPM during early withdrawal and in FSS during protracted withdrawal. In EPM locomotion was decreased, with males showing a tendency toward reduced distance travelled and speed. Previous studies have demonstrated that optogenetically activating PBN(CGRP) neurons during an actively threatening context (i.e., chasing robot) enhanced speed in both sexes (Pyeon et al., 2025). Thus, we expand the role of PBN(CGRP) on speed from an active avoidance context under alcohol‐naïve conditions to a passive avoidance context after alcohol-exposure selectively in males. Additionally, we demonstrate that PBN(CGRP)-induced changes in locomotion also extends to females albeit on immobility during a stress context in protracted withdrawal. At baseline males and females displayed sex-specific differences in immobility in FSS, an effect that has previously been noted (Kokras et al., 2015). Specifically, we demonstrate that females had increased immobility from start relative to the last day of FSS, while males displayed no adaptations in immobility. Inhibiting PBN(CGRP) neurons disrupted the adaptation in immobility in females while inducing no change in males. Given, PBN(CGRP) manipulation in females disrupted locomotor behavior with repeated stress exposure and not the initial stress exposure suggests PBN(CGRP) neurons play a role in inducing a shift from active to passive stress-coping (Commons et al., 2017). Interestingly, in males, despite no PBN(CGRP)-induced effect during FSS, a history of FSS is associated with dysregulated *ex vivo* PBN(CGRP)→BNST activity (Kayat et al., 2025). Therefore, in males, FSS-induced changes in PBN(CGRP) activity may blunt activity and thereby result in a floor effect when manipulating PBN(CGRP) neurons. Future studies will need to test this hypothesis in addition to investigating the effect of FSS on *ex vivo* PBN(CGRP)→BNST activity in females. PBN(CGRP)-induced changes in freezing and immobility, have previously been shown in threat contexts in both sexes (Palmiter, 2018; Bowen et al., 2020; Pyeon et al., 2025) but not during FSS or alcohol-exposed conditions. For example, ablating PBN(CGRP) neurons reduces freezing in response to foot shock (Han et al., 2015). Similarly, optogenetically activating PBN(CGRP) neurons without external stimuli induces freezing and immobility behavior (Bowen et al., 2020; Han et al., 2015; Pyeon et al., 2025). Although these previous studies and the present study targeted PBN(CGRP) neurons and not CGRP, the locomotor effects induced by PBN(CGRP) manipulations are in line with the role of CGRP, as targeting CGRP via ICV induces changes in locomotion (Schorscher-Petcu et al., 2009). However, it is possible that the locomotor effects may be due to other non-CGRP mechanisms. The CNO dose used to manipulate PBN(CGRP) neurons is within the range that effectively activates DREADDs without off-target behavioral effects in anxiety, reward, and alcohol-related assays (Jaramillo et al., 2020; Jendryka et al., 2019; Aomine et al., 2024; Jaramillo et al., 2018). Additionally, repeated administration of a low CNO dose does not change locomotor behavior (Tran et al., 2020), which suggest that the locomotor effects induced by repeated CNO administration in the present study are due to PBN(CGRP)-manipulations and not off-target CNO effects. However, given that CNO can be back-metabolized to clozapine, the present findings showing CNO-induced locomotor effects may be due to clozapine. Specifically, clozapine derived from CNO can act as an antagonist on adrenergic receptors (e.g., α1) (Jendryka et al., 2019), which have been shown to underlie locomotor activity in withdrawal (Becker et al., 2012) and thereby may underlie sex-specific functional responses during CIE withdrawal (Munier et al., 2022). Overall, when taken with previous work, the sex-specific effects on locomotion suggest a role for PBN(CGRP) during passive avoidance only after alcohol exposure in males. Conversely, after alcohol exposure, the role of PBN(CGRP) is specific to repeated stress and not passive avoidance context in females. Thus, PBN(CGRP) does not globally affect locomotor responses in both sexes after alcohol exposure and is instead context dependent or specific to withdrawal time. Thereby, the data suggest that decreasing PBN(CGRP) neuron excitability is sufficient to modulate locomotion following alcohol-exposure in a sex-specific manner.

Despite chemogenetic PBN(CGRP) manipulations not affecting anxiogenic behavior in naïve and alcohol-exposed conditions, a history of PBN(CGRP) inhibition during stress had subsequent effects on negative affect in both sexes. Specifically, approach frequency was decreased in NSFT, suggesting increased avoidance of the center, an anxiety-like phenotype. Speed was also decreased in males, albeit there was a trend toward decreased distance travelled and no effect on immobility, suggesting that motor function was intact. Therefore, the behavioral changes in this approach-avoidance conflict assay that was induced by inhibiting PBN(CGRP) neurons during stress imply a change in threat assessment. Given that NSFT encompasses a food component, an alternative explanation is that the change in behavior is indicative of a change in motivation. This is, in part, supported by a trend toward decreased food consumption post-testing. A decrease in motivation can be indicative of a depressive phenotype. Interestingly, clinical studies have implicated a role for CGRP in depression, as patients with major depressive disorder express higher CGRP plasma levels (Hartman et al., 2006; Mathe et al., 1994). Moreover, the PBN may underlie depressive phenotype, as optogenetically activating projections from the PBN to the VTA can induce depressive-like behavior in the tail suspension test (Zhang et al., 2021). Therefore, future testing using alternative assays to test for anxiety-like and depressive-like behaviors will help disentangle the effects of PBN(CGRP) as motivation and/or anxiety. Interestingly, when actively inhibiting PBN(CGRP) neurons, the locomotor effects were accompanied by a trend towards sex-specific effects on time spent in EPM arms during early withdrawal. However, it was only with repeated PBN(CGRP) manipulations during protracted withdrawal that the anxiogenic role of PBN(CGRP) neurons was revealed. Thereby, these findings suggest that PBN(CGRP) manipulations in withdrawal induce neuroadaptations that subsequently drive anxiety-like behavior. It is possible that inhibiting PBN(CGRP) neurons resulted in the recruitment of other brain regions (e.g., BNST, CeA) that drove the change in NSFT behavior. In the present study the PBN(CGRP) manipulations occurred in EPM and FSS, thus it is not known if manipulating PBN(CGRP) neurons in the absence of testing is sufficient to induce the effects on negative affect in NSFT. Therefore, the recruitment of other brain regions or neuroadaptations in the PBN(CGRP) may require the stressor in order to be recruited.

To our knowledge the present study is the first to demonstrate that manipulating PBN(CGRP) neurons altered a subsequent anxiety-like behavior. The role of PBN(CGRP) neurons in modulating subsequent behavior is in line with its role in fear learning. For example, optogenetically activating PBN(CGRP) neurons induced immobility in the absence of external stimuli, and pairing optogenetic PBN(CGRP) activation with a cue or context resulted in conditioned freezing behavior (Bowen et al., 2020; Han et al., 2015). Additionally, optogenetic photoinhibition or ablation of PBN(CGRP) neurons decreased freezing during a foot shock and subsequent cue or context conditioned freezing behavior (Bowen et al., 2020; Han et al., 2015). Similarly, during an actively threatening context, optogenetically activating PBN(CGRP) neurons enhanced speed during conditioning and recall (Pyeon et al., 2025). While we did not test associative learning, manipulating PBN(CGRP) neurons during stress similarly affected a later outcome, with an anxiety-like context rather than fear-related. Thus, manipulating PBN(CGRP) neurons can generate later behavioral changes. Our findings that inhibiting PBN(CGRP) neurons during stress increases anxiety-like behavior in withdrawal further implicates that stress- and anxiety-contexts recruit PBN(CGRP) neurons, which may be protective against the later behavioral effects of stress. A previous study supports the protective role of CGRP in stress, as ICV CGRP administration prior to chronic stress exposure blocked stress-induced depressive-like behavior (Hashikawa-Hobara et al., 2015). Altogether, our data imply that both sexes are sensitive to PBN(CGRP) manipulations during stress in protracted withdrawal, and the recruitment of PBN(CGRP) neurons during stress exposure may be protective in a later approach-avoidance context.

Our findings indicating that PBN(CGRP) manipulations only had an effect in males on locomotion in a passive-avoidance context in early withdrawal and in females during a stress context in protracted withdrawal, suggest that PBN(CGRP) neurons are differentially affected in both sexes across withdrawal. Moreover, despite the sex-specific effects of inhibiting PBN(CGRP) neurons, the history of manipulations induced a negative affective state in both sexes. Interestingly, our findings in alcohol-naïve conditions show that there are no sex-specific differences in CGRP expression within the PBN neurons or fibers in the BNST. While not tested directly in our study, our behavioral findings suggest that alcohol-exposure gives rise to sex-specific differences in PBN(CGRP) neuron expression and/or activity resulting in distinct locomotor responses albeit the same negative affective behavior. To our knowledge no study has analyzed if alcohol directly induces activity in PBN(CGRP) neurons. Therefore, changes in PBN(CGRP)-induced behavior may be indirectly induced by alcohol exposure such that they are instead driven by alcohol’s effect on other brain regions (e.g., BNST). Future studies will need to determine if these CGRP-associated changes are due to direct recruitment by alcohol. Additionally, future studies in which PBN(CGRP) neurons are ablated (e.g., using diphtheria toxin in CGRP-DTR^GFP^ mice) will demonstrate if the changes in behavior seen after alcohol exposure are sex-specific and dependent on neuroadaptations in PBN(CGRP) neurons. Previous work supports the theory of sex-specific neuroadaptations in PBN(CGRP) neurons, as CGRP signaling shows sex-specific changes in disease states that change over time (Presto and Neugebauer, 2022; Uchida et al., 2021). For example, in a neuropathic pain model, CGRP mRNA expression in the CeA was significantly upregulated in males during the acute phase and in females during the chronic phase (Presto and Neugebauer, 2022). Moreover, given comorbidity between pain and anxiety-like behavior, and the role of PBN(CGRP) in both contexts suggests overlap. Although the present study did not measure pain phenotypes, others have demonstrated that repeated chemogenetic activation of PBN(CGRP) neurons subsequently produces increased locomotion and anxiety-like behavior that was associated with a pain phenotype (Condon et al., 2024). Therefore, the contribution of pain to the behavioral phenotypes found in this study cannot be ruled out. Moreover, given that CIE can induce hyperalgesia and allodynia during acute withdrawal (Robins et al., 2019) further suggests that the PBN(CGRP)-induced changes in locomotion may be associated with a change in pain perception. Therefore, future studies will need to determine if PBN(CGRP)-induced negative affect is accompanied by pain phenotypes during CIE withdrawal. Taken together, our data indicate alcohol-exposed males and females respond differently to active PBN(CGRP) manipulations in distinct contexts during withdrawal. However, both males and females are sensitive to the long-term changes induced by PBN(CGRP) manipulations during withdrawal.

## 5 Conclusion

CGRP-targeted therapeutics are being considered for alcohol-induced headaches due to their efficacy in treating migraines (Hakim, 2025; Narouze, 2025). Given that negative affect, motivation, and stress-exposure can have detrimental effects during alcohol withdrawal in patients with AUD, the use of CGRP treatments in this context requires more research. Considering our findings, inhibiting CGRP-associated signaling in withdrawal may be disadvantageous for females, as it may actively impeded with stress-coping which can increase vulnerability to relapse (Sinha, 2001). Moreover, repeatedly inhibiting CGRP-associated signaling during stress exposure in withdrawal may be anxiogenic in both males and females. However, our data are specific to inhibition of PBN(CGRP) neurons. Thus, CGRP inhibitors may suppress or recruit signaling in such a way that negative affect or stress-coping is not worsened in AUD. Therefore, future studies using CGRP antagonist or monoclonal antibodies delivered either systemically or in the PBN will help further inform how these behaviors associate with direct CGRP manipulations. Moreover, given the limited number of subjects in our study, future studies are needed to further disentangle how CGRP inhibition affects behavior. Additionally, it will be of interest to investigate how PBN(CGRP) manipulations affect CGRP serum levels, as they will provide a better understanding of how peripheral CGRP signaling associates with behavior during withdrawal. In general these findings provide caution for the use of CGRP inhibitors as a pharmaceutical treatment for AUD, while lending credence to the differential effects treatments can have on both sexes.

## Data Availability

The raw data supporting the conclusions of this article will be made available by the authors, without undue reservation.
